# Feeder insects differ in passage of coccidian oocysts in captive reptiles

**DOI:** 10.1017/S0031182025101005

**Published:** 2025-11

**Authors:** Michal Berec, Gabriela Totušková, Jakub Žahourek, Jana Kvičerová, Irena Šetlíková

**Affiliations:** 1 Faculty of Science, University of South Bohemia, České Budějovice, Czech Republic; 2 Department of Parasitology, Faculty of Science, University of South Bohemia, České Budějovice, Czech Republic; 3 Department of Zoology, Faculty of Science, Charles University, Prague, Czech Republic

**Keywords:** *Blaptica dubia*, captive breeding, *Choleoeimeria pogonae*, Coccidia, *Gryllus assimilis*, *Isospora amphiboluri*, passive vector, *Pogona vitticeps*, Z*ophobas morio*

## Abstract

Coccidia (Apicomplexa) may have a devastating effect on captive reptiles, particularly chameleons and bearded dragons (*Pogona vitticeps*). This study investigated the potential of three common feeder insects – the Argentine cockroach (*Blaptica dubia*), the banana cricket (*Gryllus assimilis*) and the superworm larva (*Zophobas morio*) – to act as passive vectors for coccidian oocysts, specifically *Isospora amphiboluri* and *Choleoeimeria pogonae*, common enteric parasites of captive bearded dragons. Faecal samples from experimentally infected bearded dragons were fed to the insects to assess the passage of viable oocysts through their digestive tracts. *Gryllus assimilis* exhibited the highest passage rates for both coccidia, followed by *B. dubia; Z. morio* showed the lowest rates. However, only *G. assimilis* passed both *I. amphiboluri* and *C. pogonae* at a significantly higher rate than *Z. morio*. These findings suggest that feeder insects, particularly crickets, can act as mechanical vectors for coccidia, highlighting the importance of strict hygiene protocols in reptile keeping minimizing the risk of parasite transmission.

## Graphical abstract

**Figure:**
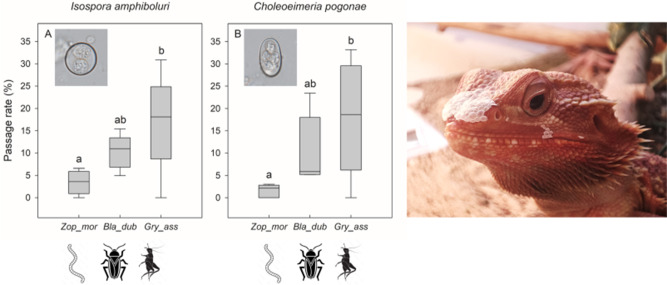

## Introduction

The popularity of keeping reptiles as pets and in zoological collections has undergone a significant increase in recent decades worldwide. This trend encompasses a diversification of the species kept and a substantial rise in the number of individual reptiles housed in a captive environment (Robinson et al. 2015; Auliya et al. 2016). While this growing interest reflects a fascination with herpetofauna, it also presents unique challenges related to animal husbandry and health management.

In captivity, reptiles are often exposed to environmental conditions that differ considerably from their natural habitats. These artificial settings can, therefore, impose a variety of non-natural stressors, such as spatially restricted enclosures, elevated population densities, frequent human interaction during maintenance and handling, and the potential for compromised hygiene protocols (Pasmans et al. 2017; Azevedo et al. 2021). The cumulative effect of these stressors may significantly impact the physiological well-being of captive reptiles, increasing their susceptibility to various health problems. Among these, infectious diseases constitute a significant concern, particularly parasitic infections, which represent a highly prevalent category (Walden et al. 2020). The proximity of reptile individuals within enclosures can facilitate the transmission of both ecto- and endoparasites, leading to morbidity and, in severe cases, mortality (McAllister et al. 1995; Modrý and Koudela 1995; Sloboda and Modrý 2006). Therefore, effective parasite management is paramount for maintaining captive reptile populations’ health and welfare.

Endoparasites encompass diverse taxa, each with its own life cycle and pathogenic potential (Walden et al. 2020). Within this group, coccidia (Apicomplexa: Eimeriidae) are frequently identified as common enteric parasites in captive reptiles (Wolf et al. 2014; Schmidt-Ukaj et al. 2017; Šlapeta et al. 2017; Walden et al. 2020). These obligate intracellular protists undergo direct life cycles, often involving the shedding of environmentally resistant oocysts in the host’s faeces, which can contaminate the environment and lead to further infections through ingestion. Specific genera and species of coccidia are recognized for their high pathogenicity (Modrý and Koudela 1995; Sloboda and Modrý 2006; Roberts et al. 2013; Szczepaniak et al. 2016; Walden and Mitchell 2021), capable of causing significant intestinal damage, malabsorption of nutrients, dehydration, and ultimately, increased susceptibility to secondary infections. Furthermore, the prevalence of specific coccidian species can be notably high in captive reptiles, potentially due to the confined living conditions and opportunities for faecal-oral transmission (Hallinger et al. 2019).

Coccidia are frequently identified in various reptile taxa, particularly in bearded dragons (*Pogona* spp.). The most common coccidian genera in these lizards include *Isospora* and *Choleoeimeria*, with *Isospora amphiboluri* and *Choleoeimeria pogonae* being frequently reported species (Papini et al. 2011; Stöhr et al. 2020; Duszynski 2021; Guardone et al. 2024). These parasites primarily inhabit the gastrointestinal tract and can cause significant morbidity (Hallinger et al. 2019). Given the potentially severe consequences of coccidial infections for the health and longevity of captive reptiles, a comprehensive understanding of their transmission modes and identifying potential risk factors are essential for developing effective preventative and control strategies. While direct faecal-oral transmission between reptile hosts is a well-established route (Chinnadurai and DeVoe 2009), indirect transmission may also play a significant role (Walden et al. 2020) and warrant detailed investigation. One such potential route involves live feeder insects, a common dietary item for many omnivorous, insectivorous and carnivorous reptile species in captivity. Feeding these insects in large numbers creates a significant risk of contact with coccidian oocysts in their environment. Consequently, unattended insects could consume infected faecal material and act as passive mechanical vectors, facilitating the parasite’s spread.

Therefore, this study aims to address a critical knowledge gap using the experimental evaluation of the capacity of three commonly fed insect taxa – the Argentine cockroach (*Blaptica dubia*), the banana cricket (*Gryllus assimilis*), and the larval stage of the superworm (*Zophobas morio*) – to act as mechanical vectors for monoxenous coccidia. Specifically, we sought to determine whether the oocysts of these parasites can survive passage through the digestive tract of these insects, thereby posing a potential risk of transmission to reptiles upon ingesting the contaminated feeder insects. The findings of this research contribute to the knowledge on the possible role of feeder insects in the epidemiology of coccidial infections in captive reptiles and to the development of enhanced biosecurity measures in reptile husbandry.

## Materials and methods

### Collection of coccidia-positive samples for experimental infection

Coccidia-positive faecal samples were collected from a small household breeding group of three bearded dragons (*Pogona vitticeps*) with a confirmed coccidial infection. Samples were collected into 50 mL tubes containing a 4% (w/v) potassium dichromate preservation solution (K_2_Cr_2_O_7_) as soon as possible after defecation and stored at 4°C for a maximum of two weeks before processing. Before use in the experimental inoculation, faecal samples were cleaned by centrifugation: the faeces were placed in a centrifuge tube, brought to a total volume of 12 mL with tap water, and centrifuged at 1540× *g* for 10 minutes. This procedure was repeated two to three times to ensure complete removal of the K_2_Cr_2_O_7_.

### Determination of coccidian species from faecal samples

Species identification of coccidia in the collected samples was performed using morphological and molecular approaches. Morphological determination of the coccidian species was based on comparisons with previous descriptions of coccidia identified in members of the family Agamidae (Supplementary Table 1). Oocysts were morphologically and morphometrically analysed using light microscopy (Olympus BX53 microscope) at 10 × 40 magnification, equipped with a DP-73-1-51 high-resolution, cooled digital camera, and Olympus cellSens Standard 1.13 imaging software (Olympus, Tokyo, Japan).

**Figure 1.**
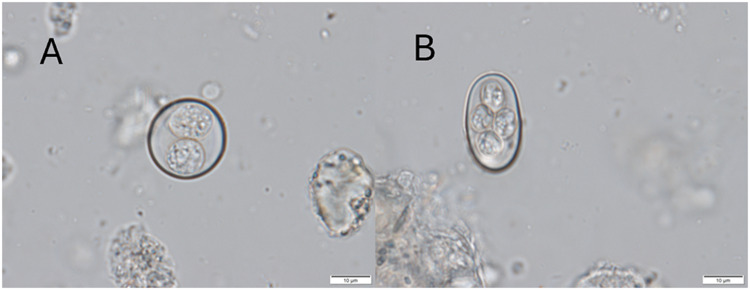
Reptilian coccidia of morphotypes *Isospora* (A) and *Eimeria* (B) (photo: Jakub Žahourek).

Two distinct species of coccidia were microscopically identified in faecal samples of the bearded dragons (Figure 1): *I. amphiboluri* and *C. pogonae*. The species identity was further verified by Sanger sequencing of the partial 18S rDNA and COI genes (for detailed methodology, see Trefancová et al. 2021).

### Quantification of oocysts in the faecal samples

The number of oocysts in the samples was determined using the oocysts per gram (OPG) method by counting in a standard haematological Bürker counting chamber (Kváč et al. 2007). Centrifuged faecal samples were weighed in grams to two decimal places. One mL of phosphate-buffered saline (PBS) solution was added to 1 *g* of the sample. The mixed suspension of the sample and buffer was loaded onto the Bürker chamber’s counting surface using a Pasteur pipette. Coccidian oocysts were counted under a light microscope at a magnification of 200× in a total of 50 small squares (25 consecutive small squares in each of the two counting grids of the chamber). According to Bürker’s rule, all oocysts lying on or touching a square’s lower or left side were counted, while those lying on or touching the upper or right side were excluded (Gunetti et al. 2012; Kudlová 2021). The OPG was then calculated using the following formula:

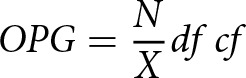


where *OPG* = number of coccidian oocysts per gram of faeces; *N* = average number of coccidian oocysts per small square; *X* = weight of faeces in grams; *df* = dilution factor (here, multiplied by 2); *cf* = coefficient converting volume of the small square (surface: 0·0025 mm^2^ and depth: 0·1 mm) per 1 mL (here multiplied by 2500).

### Experimental inoculation of feeder insects

The cleaned faecal sample, for which OPG had been determined, was again weighed and mixed with fruit purée in a 1:1 ratio, which served as an attractant to enhance sample ingestion. The mixture was then offered to the fasted experimental insects. Commonly available feeder insect species from commercial breeding facilities were used for the experiment. The adults of Argentine cockroach (*B. dubia*) represented the order Blattodea (cockroaches), the adults of banana cricket (*G. assimilis*) were selected from the order Orthoptera (grasshoppers, crickets, locusts and allies), and the large larval stage of the superworm (*Z. morio*) represented the order Coleoptera (beetles). To ensure the absence of coccidia, three individuals of each feeder insect species were randomly selected and examined prior to the experiment, using the procedure described above.

Before sample administration, the insects were deprived of food and water for five days to ensure complete gut evacuation. Subsequently, five individuals of each insect species were moved into individual plastic test boxes (10 cm in diameter, 7 cm in height). These boxes contained fruit purée mixed with a faecal sample, which was supplied *ad libitum* for two days. A pilot study, using dissection, had previously established that this duration was enough to fully fill the insects’ digestive systems.

### Dissection of feeder insects

After offering the parasite infected puree, the insects in each of the five experimental boxes were euthanized with ethanol, and a suspension was prepared from their intestines (posterior half of the midgut and colon). Intestinal material was weighed and centrifuged for 10 minutes at 1540× *g*. After the supernatant was discarded, the sediment was resuspended in 1 mL of PBS solution per gram of sample, and the OPG was then determined in this suspension as explained above. The OPG in the insect’s intestine was quantified by analysing three subsamples from each individual. Subsequently, the passage rate was calculated as the proportion of the insect’s intestinal OPG, expressed as a percentage, with the bearded dragon’s faecal OPG as the 100% reference point.

### Statistical analysis

We tested the differences in passage rates of specific parasites among feeder insects (independent variable) using a Kruskal–Wallis test followed by non-parametric post-hoc comparisons. The analyses were performed in Statistica 14.0 and visualized in SigmaPlot 14.0.

## Results

**Figure 2.**
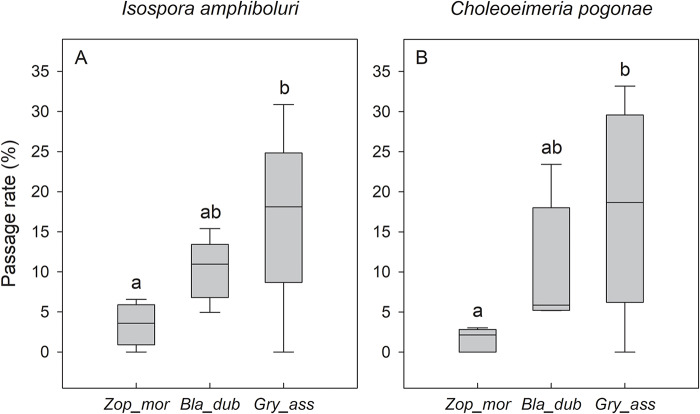
Passage rate (%) of *Isospora amphiboluri* (A) and *Choleoeimeria pogonae* (B) in different species of feeder insects: *Zophobas morio* (*Zop_mor*), *Blaptica dubia* (*Bla*_*dub*) and *Gryllus assimilis* (*Gry_ass*). Boxplot: median ± quartiles and 10th–90th percentiles are shown.

The experimental results confirmed the capacity of *B. dubia, G. assimilis* and *Z. morio* to pass viable oocysts of coccidia of the genera *Isospora* and *Choleoeimeria* through their gastrointestinal tracts. The highest passage rates for both coccidian species were observed in the digestive tract of the cricket *G. assimilis*, with an average passage of 18% (IQR: 17–19%) for *I. amphiboluri* and 19% (IQR: 12–26%) for *C. pogonae*. The cockroach species *B. dubia* exhibited an intermediate level of coccidian passage, with an average of 11% (IQR: 9–11%) and 6% (IQR: 5–13%) for *I. amphiboluri* and *C. pogonae*, respectively. The lowest passage rates of coccidian oocysts were detected in the larval stage of the beetle *Z. morio*, with an average passage of 4% (IQR: 2–5%) for *I. amphiboluri* and only 2% (IQR: 0–3%) for *C. pogonae* (Figure 2).

From a statistical standpoint, the passage of both coccidian species *I. amphiboluri* and *C. pogonae* differed significantly among the insect species tested (*H* = 6·15, *p* = 0·046 and *H* = 6·79, *p* = 0·034, respectively). Post-hoc analysis revealed specific, significant variations in transmission rates between the groups. For *I. amphiboluri*, the passage rate was significantly higher in *G. assimilis* than in *Z. morio* (*p* = 0·049) (Figure 2A). For *C. pogonae*, the passage rate was also significantly higher in *G. assimilis* when compared to *Z. morio* (*p* = 0·049) (Figure 2B).

## Discussion

Morphological analysis of faecal samples of bearded dragons revealed two distinct coccidia, identified as *I. amphiboluri* and *C. pogonae*. These two species are commonly found in bearded dragons (Schmidt-Ukaj et al. 2017; Guardone et al. 2024), and both are potentially pathogenic to them. Infection with *I. amphiboluri* can harm the intestinal mucosa, potentially leading to decreased absorption of nutrients and fluids, and, consequently, diarrhoea (Kim et al. 2002; Walden and Mitchell 2021). Although often asymptomatic in adult bearded dragons, this parasite can cause severe illness and death in juveniles, possibly due to ongoing reinfection if its oocysts persist in the environment (Walden and Mitchell 2021). Likewise, infection with *Choleoeimeria* in bearded dragons has been linked to emaciation and dehydration (Szczepaniak et al. 2016). Severe infections with *Choleoeimeria* can result in gallbladder enlargement, localized thickening of the gallbladder wall, the formation of small gallstones and/or debris, blockage of the bile ducts, and, ultimately, death (Stöhr et al. 2020). Therefore, increased husbandry hygiene and a strict feeding protocol are highly desirable in these cases. The potential for oocyst transmission via feeder insects is also a significant consideration.

Our experiments revealed that three distinct feeder insect species could transmit viable oocysts of both coccidian genera, exhibiting notable variations in their passage rates. Specifically, *G. assimilis* exhibited higher passage of both *I. amphiboluri* and *C. pogonae* than *Z. morio*. On the other side, *B. dubia* had a similar passage rate to the other feeder insect species for both parasites.

Given the very limited passage of oocysts through their digestive systems, feeding terrarium animals with *Z. morio* larvae might pose the lowest risk of coccidial infection. Factors influencing such a low passage rate could include their highly efficient digestion and gut microbiota, which some studies suggest enable them to break down materials like polystyrene and other commercial plastics (Yang et al. 2020; Pham et al. 2023; Sun et al. 2025). Consequently, their digestive processes and feeding habits might disrupt the oocyst structure, damaging the oocyst wall, which is crucial for survival outside a host (Mai et al. 2009). However, coccidian oocyst passage rates likely vary among different species of tenebrionid beetles. For example, Dunford and Kaufman (2006) observed the transmission of several pathogens, including coccidia of the genus *Eimeria*, by the lesser mealworm (*Alphitobius diaperinus*) in poultry houses, where this specific beetle is considered a pest.

The role of cockroaches in parasite transmission is well-documented (Tatfeng et al. 2005; Alzain 2013; Oyeyemi et al. 2016). Jarujareet et al. (2019) further demonstrated the capacity of American cockroaches (*Periplaneta americana*) in commercial chicken farms to harbor and transmit oocysts of highly pathogenic species *Eimeria tenella* over an extended period. Their study demonstrated that viable oocysts persisted in the cockroach gut for at least four days post-ingestion and remained infective even after excretion. In our results, the passage of both parasite species was higher than that of *Z. morio*, but this was not statistically proven. This might be attributed to the unexpectedly high variability in individual passage rates.

*Gryllus assimilis* was the species with the highest number of passaged oocysts among the three species studied; however, it was also the species with the highest variability of passage rate. Crickets are known carriers of coccidia (Rowe et al. 2024) and are frequently identified as a source of coccidiosis in livestock (Day and Rowe 2002; Szelei et al. 2011; Gałęcki and Sokół 2019). Gałęcki and Sokół (2019) specifically attribute the presence of *Isospora* spp. in edible insects to poor hygiene standards on insect farms. Our findings suggest that these inadequate hygiene practices can also facilitate the further spread of infections through the digestive tract of crickets. However, the high variability in oocyst passaging ability would merit further research.

Intestinal transit time and the capacity for coccidian oocyst degradation may have significantly contributed to the observed differences. When focusing on the time required for food to reach the rectum, the relative digestive speeds among insects become apparent (due to the absence of precise digestion time data for the three species employed in this study, we used information from the most closely related species available). The lesser mealworm (*A. diaperinus*) exhibits slow overall passage to the end of the digestive tract, with mean transit times ranging from about 132 to 186 minutes and maximum times reaching up to 300 minutes (Zheng et al. 2012). Similarly, the German cockroach (*Blattella germanica*) needs around 300 minutes for food to reach its rectum after a 2-day starvation period (Day and Powning 1949). In contrast, the starved two-spotted cricket (*Gryllus bimaculatus*) shows the fastest transit, with food being digested in approximately 80 minutes (Woodring and Lorenz 2007). Thus, a higher passage time indicates a higher passage rate.

In conclusion, coccidiosis can be a serious health problem in intensively reared reptiles such as bearded dragons and chameleons. We confirmed the ability of all three species of feeder insects to pass viable oocysts of two coccidian species that are pathogenic in reptile breeding. For the first time, we demonstrated different passaging rates under the same conditions across all three insect species. The observed variations in oocyst passage may stem from differences in the ability of different insect species to harbour and shed parasitic stages. Regardless of the experiment limitations, the consistent finding of the highest percentage of passed oocysts in crickets underscores the critical importance of maintaining stringent hygiene protocols during feeding, particularly when using crickets as a primary food source for captive reptiles. This is not a reason to relax hygiene practices; it reinforces the need for increased awareness and preventative measures to minimize the risk of parasite transmission. Based on our results, we recommend feeding feeder insects more frequently and in smaller quantities. The key rule is to ensure feeding insects never consume droppings from infected captive reptiles. This ensures their rapid consumption while simultaneously prevents their contact with reptile faeces. For an adult bearded dragon, this can be approximately five crickets every other day.

## Supporting information

Berec et al. supplementary material Berec et al. supplementary material

## Supplementary material

The supplementary material for this article can be found at https://doi.org/10.1017/S0031182025101005.
