# Supplementary material for: Feeder insects differ in passage of coccidian oocysts in captive reptiles
Source: Parasitology. 2025 Oct 27;152(13):1381–6. doi: 10.1017/S0031182025101005 (PMC12917416; doi:10.1017/S0031182025101005)
Supplement: Berec et al. supplementary material [file S0031182025101005sup001.docx]

Supplementary Table

Table 1: Coccidia of “*Isospora* morphotype” and „*Choleoeimeria* morphotype“ so far described from agamid lizards (Agamidae). Abbrevitations: SB, Stieda body; sSB, substieda body; pSB, parastieda body; N.D., not described.

| **Coccidia** | | **Oocyst** | | | | | | **Sporocyst** | | | | |
| --- | --- | --- | --- | --- | --- | --- | --- | --- | --- | --- | --- | --- |
| **Species** | **Host** | **Shape** | **Size [µm]** | **Wall** | **Polar granule** | **Residuum** | **Micropyle** | **Shape** | **Size [µm]** | **SB** | **sSB/ pSB** | **Residuum** |
| *Isospora ameivae*  Carini (1932), Lainson and Paperna (1999) | *Ameiva ameiva* | subspherical | 17.2 × 15.0  (15.5-18.5 × 14.1-15.5) | smooth, colourless,  1 layer | present, 1 piece, ovoidal | absent | absent | pear-shaped | 12.1 × 7.5  (11.8-13.3 × 7.4-8.1) | present, barely apparent | both absent | present, massive; fine granules and a few larger globules |
| *Isospora amphiboluri*  Cannon (1967),  McAllister et al. (1995) | *Pogona barbata*,  *Pogona henrylawsoni*, *Pogona vitticeps* | subspherical to cylindrical | 25.3 × 25.1  (23-26 × 23-26) | smooth,  2 layers | absent | absent | absent | cylindrical | 17.0 × 11.4  (16-18 × 11-12) | present | sSB present/pSB absent | present, compact, subspherical, composed of numerous coarse granules |
| *Isospora amphiboluri*  Liu et al. (2021) | *Ctenophorus nuchalis* | cylindrical | 24.2 × 23.9  (23.0-26.5 × 22.4-25.9) | smooth, yellowish,  2 layers | absent | absent | absent | lemon-shaped | 15.7 × 10.2  (15.2-18 × 8.9-11.2) | present, small | sSB present/pSB absent | present, finely dispersed, granular |
| *Isospora bronchocelae*  McQuistion et al. (2001) | *Bronchocela cristatella* | subspherical to ovoidal | 25.2 × 22.3  (20-28 × 19-24) | 2 layers, outer striated and pitted | present, 1 piece (1-2 globules stuck together, or a splinter-shaped mass) | absent | absent | ovoidal | 14.8 × 9.9  (13-16 × 9-10) | present, broad, dome-like | sSB present, large, fan-like/  pSB perhaps present | present, coarse granules clustered in an amorphous mass |
| *Isospora cannoni* Finkelman and Paperna (1994) | *Diporiphora australis* | subspherical | 22.8 × 24.8  (20.0-25.0 × 22.5-27.5) | smooth | absent | absent | absent | ovoidal | 14.7 × 10.2  (14-15.5 × 10-11.5) | present | sSB present, conspicuous/pSB perhaps present | present |
| *Isospora caryophila* Rogier and Colley (1976) | *Gonocephalus grandis* | subspherical to ellipsoidal | 23.5 × 21.9  (21-30 × 18-29) | 1 layer | present, 1 piece | N.D. | N.D. | ovoidal | 13.2 × 8,2  (9-15 × 7-10) | present, very small | sSB absent/pSB perhaps present | present, scattered |
| *Isospora choochotei* Finkelman and Paperna (1994) | *Calotes mystaceus* | subspherical to cylindrical | 29.3 × 29.5  (24.0-32.0 × 28.0-32.5) | probably 1 layer | absent | absent | absent | ovoidal | 16.5 × 11.2  (15.5-18 × 11) | present | sSB absent/pSB perhaps present | present |
| *Isospora deserti* Finkelman and Paperna (1994) | *Trapelus mutabilis, Trapelus pallidus* | spherical | 27.7 × 27.7  (25-28 × 25-28) | smooth | absent | absent | absent | ovoidal | 16.1 × 10.7  (14-17.5 × 10-11) | present | sSB present/pSB perhaps present | present |
| *Isospora farahi* Mihalca et al. (2009) | *Agama rueppelli* | spherical or subspherical | 29.1 × 28.8  (26-31 × 26-31) | smooth, brownish,  2 layers | absent | absent | absent | oval | 16.6 × 11.4  (15-18 × 11-12) | present, discoid | sSB present, globular/pSB absent | present, medium-sized granules irregularly scattered among sporozoites |
| *Isospora gonocephali*  Maupin et al. (1998) | *Gonocephalus grandis* | subspherical to ovoidal | 22.3 × 18.7  (19-25 × 17-23) | 2 layers, outer spotted | present, 1 piece | absent | N.D. | almond-shaped | 13.5 × 9.2  (12-15 × 8.5-10) | present, wide, dome-like | sSB present/pSB perhaps present | present, composed of non-uniform coarse granules in an amorphous cluster |
| *Isospora lacertae* Saum et al. (1997) | *Calotes versicolor* | subspherical to ovoidal | 28.1 × 26.5  (23-31 × 23-28) | 2 layers, outer smooth, but striped | absent | N.D. | N.D. | ovoidal | 14.6 × 10.3  (13.0-15.0 × 7.0-11.0) | present, dome-like | sSB present, squared/pSB perhaps present | present, subspherical, coarse granule cluster |
| *Isospora phrynocephali* Ovezmukhammedov (1971) | *Phrynocephalus helioscopus* | spherical | 26.2 × 26.2  (24.3-27.0 × 24.3-27.0) | N.D. | N.D. | N.D. | N.D. | subspherical | 14.7 × 9.2  (13.5-18.9 × 8.1-13.5) | N.D. | N.D. | N.D. |
| *Isospora rayi* Mandal (1966) | *Ptyctolaemus gularis* | spherical | 26.3 × 26.3  (25.5-27.4 × 25.5-27.4) | N.D. | N.D. | N.D. | N.D. | navicular | 15.4 × 8.6  (14.5-16.3 × 9.5-10.5) | N.D. | N.D. | N.D. |
| *Isospora rustamovi*  Ovezmukhammedov (1971) | *Phrynocephalus reticulatus* | spherical | 26.2 × 26.2  (18.9-32.4 × 18.9-32.4) | N.D. | N.D. | N.D. | N.D. | pear-shaped | 16.5 × 11.7  (13.5-18.9 × 10.8-13.5) | N.D. | N.D. | N.D. |
| *Choleoeimeria pogonae* comb. nov.  Szczepaniak et al. (2016) | *Pogona vitticeps* | ellipsoidal | 28.4 × 16.8 (25.6-32.3 × 13.6-21.4) | smooth, 2 layers | absent | absent | absent | ovoidal | 12.5 × 6.6  (9.4-14.7) × (5.3-7.4) | absent | absent | present |
| *Choleoeimeria pogonae*  Yang and Brice (2016) | *Pogona minor minor* | cylindrical | 27.0 × 15.2  (26.0-28.3 × 14.0-16.5) | 2 layers | present, 1 piece | absent | absent | ovoidal | 10.0 × 8.5  (9.0-11.0) × (7.0-9.5) | absent | absent | N.D. |
